# Triple-Survival Stereotactic Brain Surgeries for the Intracranial Injections of Glioblastoma Stem-like Cells and Oncolytic Herpes Simplex Viruses

**DOI:** 10.3390/mps9030082

**Published:** 2026-05-31

**Authors:** Sourav Chakraborty, Connor Howard, Checo J. Rorie, Samuel D. Rabkin, Hiroaki Wakimoto, Dipongkor Saha

**Affiliations:** 1 Department of Biology, North Carolina Agricultural and Technical State University, Greensboro, NC 27411, USA; schakraborty3@aggies.ncat.edu (S.C.); choward12@aggies.ncat.edu (C.H.); cjrorie@ncat.edu (C.J.R.); 2 Brain Tumor Research Center, Department of Neurosurgery, Massachusetts General Hospital and Harvard Medical School, Boston, MA 02114, USA; rabkin@mgh.harvard.edu (S.D.R.); hwakimoto@mgh.harvard.edu (H.W.)

**Keywords:** glioblastoma, stereotactic surgery, intracranial injection, survival surgery, oncolytic herpes simplex virus

## Abstract

Glioblastoma (GBM) is an aggressive primary brain tumor associated with poor prognosis and resistance to therapy, underscoring the need for reliable preclinical models to evaluate emerging treatments. The orthotopic implantation of GBM stem-like cells (GSCs), combined with the intratumoral delivery of therapeutic agents, represents a widely used approach for modeling GBM tumor growth and studying treatment response. In particular, oncolytic herpes simplex viruses (oHSVs) have emerged as a promising strategy to selectively target malignant cells while inducing antitumor immune responses with minimal systemic toxicity. However, performing repeated survival stereotactic neurosurgeries in the same animal poses significant technical challenges. Here, we describe a comprehensive and reproducible protocol for triple survival stereotactic neurosurgery in mice. This approach involves (i) the intracranial implantation of GSCs to establish orthotopic tumors, (ii) the intratumoral delivery of oHSV using the same stereotactic coordinates, and (iii) contralateral intracranial rechallenge with GSCs to evaluate therapeutic efficacy and resistance to tumor rechallenge as a measure of immune memory. Using this protocol, consistent tumor establishment was achieved, and mice tolerated repeated neurosurgical procedures with stable postoperative recovery. Successful intracranial rechallenge in the same animal demonstrates the technical feasibility of multiple survival surgeries while minimizing procedure-related variability and complications. This method enables longitudinal assessment of tumor progression, therapeutic response, and durable memory protection within a single subject. Furthermore, this protocol provides a versatile platform for evaluating oncolytic virotherapy and other localized treatment strategies for GBM.

## 1. Introduction

Glioblastoma (GBM) is the most common primary malignant brain tumor and remains almost uniformly lethal despite standard therapies, including surgical resection, radiotherapy, and chemotherapy [1]. A major driver of treatment failure is the presence of GBM stem-like cells (GSCs), which promote tumor initiation, progression, immunosuppression, and resistance to therapy [2,3]. Consequently, orthotopic GSC-based murine GBM models provide biologically relevant platforms for evaluating novel therapeutic strategies [4,5,6].

Oncolytic viruses (OVs), particularly oncolytic herpes simplex virus (oHSV), have emerged as promising therapeutics for GBM due to their unique mechanisms of action: direct tumor cell lysis and induction of antitumor immune responses [7,8]. G47Δ, an engineered oHSV lacking ICP6, ICP34.5, and ICP47, has demonstrated clinical efficacy and was approved for recurrent GBM in Japan [9]. Further modification to express interleukin-12 (IL-12), a key regulator of antitumor immunity, enhances immune activation and therapeutic efficacy in preclinical GSC-based GBM models [10,11,12]. Importantly, oHSV-based therapies are increasingly recognized not only for their capacity to reduce tumor burden but also for their potential to generate immunological memory [8,13].

The evaluation of therapeutic efficacy and immune memory in orthotopic GBM models requires a longitudinal experimental design in which tumor establishment, treatment delivery, and tumor rechallenge are performed within the same animal. In this context, the intracranial rechallenge of tumor-free mice provides a functional readout of treatment-induced immune memory [14]. However, implementing such studies necessitates repeated stereotactic neurosurgical procedures, which present technical challenges, including maintaining targeting accuracy across surgeries, minimizing cumulative tissue damage, and ensuring animal survival and recovery. Despite the increasing use of multi-surgery approaches [10,14,15,16,17,18,19,20,21] and the availability of several stereotactic surgery protocols [22,23,24,25,26,27,28,29,30], there is currently no standardized method that integrates intracranial tumor implantation, intratumoral therapeutic delivery, and contralateral tumor rechallenge within a single reproducible workflow.

Here, we describe a comprehensive triple-survival stereotactic neurosurgical protocol that enables (i) intracranial implantation of GSCs for orthotopic tumor establishment, (ii) precise intratumoral delivery of oHSV using consistent stereotactic coordinates to minimize tissue disruption, and (iii) contralateral intracranial rechallenge to evaluate treatment-induced immune memory. By integrating these steps within the same animal, this protocol provides a robust platform for assessment of tumor progression, therapeutic response, and memory immunity in GBM.

## 2. Experimental Design

This experimental design involves repeated, precise stereotactic neurosurgery in the same animal in a Biosafety Level 2 (BSL-2) facility. The procedures for multiple survival surgeries also require the proper monitoring and maintenance of anesthesia, along with pre- and postoperative care, to minimize surgical pain and distress, reduce variation in physiological responses, and ensure animal welfare throughout the experiment.

### 2.1. Materials

- GSCs such as 005 GSCs [10,31];
- Gibco advanced DMEM/F-12 reduced serum medium (Fisher Scientific, Hampton, NH, USA, Cat. # 12634010);
- L-Glutamine (Fisher Scientific, Cat. # A2916801);
- N2 supplement (Fisher Scientific, Cat. # A1370701);
- Antibiotic-Antimycotic solution (Fisher Scientific, Cat. # 15240062);
- Epidermal Growth Factor (EGF) (Fisher Scientific, Cat. # 315-09-500UG);
- Fibroblast Growth Factor (FGF) (Fisher Scientific, Cat. # 450-33-50UG);
- Heparin (Millipore Sigma, Burlington, MA, USA, Cat. # H3149-10KU);
- OHSV G47Δ-IL12 [Note: Although G47Δ-IL12 is used here, this protocol is appropriate for intratumoral delivery of other OVs or therapeutics [32,33,34,35]];
- Styrofoam container (200 × 150 × 180 mm);
- C57BL/6 mouse (7–8 weeks old; Charles River Laboratories, Wilmington, MA, USA);
- Clorox (The Clorox Company, Oakland, CA, USA)/10% bleach;
- Ketamine HCL (from any vendor) [Note: Approval from the United States Drug Enforcement Administration (DEA) is required to purchase ketamine];
- Xylazine HCL (Fisher Scientific, Cat. # MP215830701);
- Non-Woven Gauze Sponges (2 × 2 inches; Fisher Scientific, Cat. # 22-028-559);
- 0.25% Bupivacaine (Fisher Scientific, Cat. # 503042978);
- Carprofen (Generic) Injection (Chewy, Plantation, FL, USA, SKU: 510510_-RX);
- Hydration Supplement: Sucralose (ClearH_2_O, Westbrook, ME, USA, SKU: 74-02-5022);
- Sterile Lubricant Eye Ointment (Med-Vet International, Mettawa, IL, USA, Item no. 63736-238-24);
- Model 963 Ultra Precise Small Animal Stereotaxic Instrument (David Kopf Instruments, Tujunga, CA, USA);
- Scale to measure the distance from the bregma (note: this comes with the Model 963)
- HP-1M Heating Plate (David Kopf Instruments);
- Stanfield Heat Mat—2 × 3 ft (Osborne Livestock Equipment, Osborne, KS, USA, SKU: FH-RS2B30);
- Povidone-Iodine Prep Pads (Fisher Scientific, Cat. # 06-669-70);
- Sterile Alcohol Prep Pads (Fisher Scientific, Cat. # 19090834);
- Sterile phosphate-buffered saline (PBS) (1×) (Fisher Scientific, Cat. # MT21040CV);
- Isopropyl Alcohol (Fisher Scientific, Cat. # STL364201);
- Ideal Micro-Drill (SouthPointe Surgical Supply, Coral Springs, FL, USA, Cat. # CP67-1200);
- Ideal Burr Set 0.6 mm (SouthPointe Surgical Supply, Cat. # CP60-1000);
- Hamilton 10 μL Gaslight Syringe with Cemented Needle (Cole-Parmer, Vernon Hills, IL, USA, Mfr. 80000);
- Oak Ridge Sharps Container with Slide Lid (Fisher Scientific, Cat. # 22-730-449);
- Cotton Swab (Fisher Scientific, Cat. # 22-363-163);
- Kim Wipes (Fisher Scientific, Cat. # 06-666C);
- Bone Wax 2.5 g (Fisher Scientific, Cat. # 501180260);
- Protected Disposable Scalpels Size 11 (Fisher Scientific, Cat. # 02-688-79);
- Needle holder (Fisher Scientific, Cat. # 22-445-804);
- Scissors (Fisher Scientific, Cat. # 08940);
- Forceps (Fisher Scientific, Cat. # 13-812-41);
- Ethilon Nylon Suture Green Monofilament, Size 5.0 (Med-Vet International, Cat. # G666G);
- Weight Measuring Scale (Fisher Scientific, Cat. # 01-921-101).

### 2.2. Preparation of Cells, Virus, Medications, and Anesthetizing Agents

#### 2.2.1. Preparation of 005 GSCs for Intracranial Implantation

Prepare a single-cell suspension of 005 GSCs in a 1.7 mL microcentrifuge tube at the desired concentration, which can vary depending on the experimental design and treatment goals. Here, for our survival studies, we prepare 2 × 10^4^ 005 GSCs in 3 µL 005 GSC medium [16] per mouse and keep the tube on ice. Notes: (i) If intracranial implantation of 005 GSCs is performed in 20 mice, always prepare cells for 50% more mice (i.e., 30 mice × 3 µL/mouse = 90 µL total; each 3 µL contains ~2 × 10^4^ 005 GSCs) to account for loss and to ensure that the tip of the Hamilton Syringe is always dipped under the cell suspension when withdrawing cells from the microcentrifuge tube to avoid drawing any air into the Hamilton syringe. (ii) Cells will settle to the bottom of the microcentrifuge tube, so gently flick the bottom of the tube 5–7 times with the index finger to ensure homogenous distribution immediately before withdrawing the cell suspension into the needle.

#### 2.2.2. Preparation of G47Δ-IL12 Working Stock for Intratumoral Delivery

Usually, for survival studies, a dose of 5 × 10^5^ plaque forming units (PFU)/mouse in 2 µL sterile 10% glycerol/PBS is prepared from 4 × 10^8^ PFU/mL original G47Δ-IL12 stock in a 1.7 mL microcentrifuge tube on the day of intratumoral oHSV injection. The prepared G47Δ-IL12 solution is transported to the animal facility surgical area on the day of surgery using a Styrofoam container containing ice to maintain temperature stability. Notes: (i) The virus titer in the original stock is determined before each experiment and in the working stock after the experiment to ensure experimental rigor following the oHSV titration protocol described previously [36]. (ii) As with 005 GSCs (Section 2.2.1), the oHSV stock is diluted to the final concentration and volume, depending on the experimental design and purpose. Importantly, always count 50% more mice when preparing the working solution. For example, if an intracranial oHSV injection is performed in 20 mice, the oHSV solution is prepared for 30 mice (i.e., 30 mice × 2 µL/mouse = 60 µL, i.e., 37.5 µL of 4 × 10^8^ PFU original stock + 22.5 µL 10% glycerol/PBS = 60 µL; each 2 µL contains ~5 × 10^5^ PFU of G47Δ-IL12). (iii) The injection volume can be adjusted to 3 µL/mouse, and in fact, in this protocol, we used 1 × 10^6^ pfu viral dose/mouse in a volume of 3 µL/mouse; however, a lower volume, like 2 µL, is preferred since the viscosity of the oHSV working stock solution in 10% glycerol/PBS is much lower than the 005 GSC suspension, reducing the risk that the virus solution may come out when injecting intracranially into the brain tumors.

#### 2.2.3. Preparation of Carprofen Oral Gel

An analgesic drug is provided using medicated oral gel to ensure continuous pain control before and after surgery while minimizing stress associated with repeated subcutaneous carprofen injections. Carprofen is incorporated into sucralose-based hydration gel to allow voluntary consumption and consistent drug delivery. A typical analgesic dose is 10 mg/kg/day (Table 1). For example, a 20 g mouse (0.02 kg) requires approximately 0.2 mg of carprofen per day. Under standard housing conditions with five mice per cage, the total body weight per cage is approximately 100 g, corresponding to a daily carprofen requirement of 0.2 mg × 5 mice = 1.0 mg per cage. Each mouse can consume approximately 5 mL of gel per day, so five mice will collectively consume about 25 mL of sucralose gel per day, which should contain 1.0 mg of carprofen. A standard 60 mL (2 oz) cup of sucralose gel, which typically supports up to five mice for approximately 2.5 days, will require approximately 2.4 mg of carprofen per cup. To prepare the medicated gel, as per the manufacturer’s instructions (ClearH_2_O^®^), warm the gel container in a water bath at approximately 60 °C for up to 15 min until the gel becomes fluid (Note: avoid using a microwave to prevent uneven heating). Add the calculated carprofen solution (i.e., 2.4 mg) into the liquefied gel and mix thoroughly with a spatula. Allow the gel to cool to room temperature for 30 min or refrigerate for about 15 min, to re-solidify it before placing it in the cage (one cup/cage). The medicated gel should serve as the primary source of hydration and analgesia, and it should be added to the cage at least 24 h before surgery to ensure adequate pain control and allow animals to adapt to the gel-based delivery method.

#### 2.2.4. Preparation of Ketamine/Xylazine General Anesthesia

On the day of surgery, a general anesthesia solution is freshly prepared using the following combination: 100 mg/kg Ketamine plus 10 mg/kg xylazine in sterile PBS [37]. A ketamine (100 mg/kg)/xylazine (10 mg/kg) mixture is prepared in 100 µL PBS solution/20 g mouse (i.e., 5 µL/g mouse) and the solution kept on ice (Table 1). The final injection volume is adjusted for each mouse individually based on the measured body weight, e.g., a mouse weighing 21 g requires 105 µL of the ketamine/xylazine mixture.

### 2.3. Limitations

The success of triple survival stereotactic surgery depends on multiple technical and biological factors, including, but not limited to, accurate anesthetic dosing, appropriate preoperative care, precise stereotactic positioning and head fixation, controlled skull drilling, accurate intracranial needle placement within the brain parenchyma, and proper postoperative recovery and pain management. Despite careful execution of all steps outlined below, perioperative complications may still occur. For example, based on our experience, ~1/64 mice may show prolonged or difficult recovery, likely due to cumulative surgical stress.

In addition, technical errors, such as inadvertent injection of cells into the ventricular system, can result in inconsistent or failed tumor establishment. Animal selection is also an important consideration. For instance, mice weighing less than ~18 g are generally not suitable for this protocol, as they may not tolerate the cumulative burden of multiple survival surgeries. Technical risks include uncontrolled skull drilling, which may damage the meningeal layers and lead to intracranial hemorrhage or hematoma formation. Furthermore, stereotactic neurosurgery inherently carries risks of localized pain, neurological complications, or, in rare cases, mortality associated with the procedure.

## 3. Procedure

### 3.1. Pre-Surgery Preparations: Workbench, Biosafety Cabinet, General Anesthesia, and Stereotactic Frame

#### 3.1.1. Pre-Surgery Pain Management

At least 24 h before surgery, oral carprofen gel is added to the cage (one cup/cage).

#### 3.1.2. Preparation of the Working Bench

On the day of surgery, the working bench is disinfected, where the mouse cages are kept for presurgical preparation and postoperative recovery, first with 10% bleach/Clorox, followed by wiping the bench with 70% isopropyl alcohol, and the bench allowed to dry. The Stanfield Heat Mat placed on the working bench is turned on and warmed up for 5 min. Afterward, a cage (carrying 4–5 mice, maximum) is brought onto the warmed heat mat and, using a weighing scale, the body weight of each mouse measured. Note: A heat lamp is not to be used. We often use T-cell-deficient athymic nude mice (nu/nu) to study the T-cell-dependent mechanism of oncolytic virotherapy, which lack fur and are more sensitive to temperature changes and direct heat from a heat lamp; thus, a heat lamp is not recommended.

#### 3.1.3. Induction of General Anesthesia

First, the mice are restrained by the scruff method. Briefly, the base of the tail is held and placed on a plain surface, and then the loose skin at the neck region firmly scruffed and head movement limited with the forefinger. Four to five mice (one at a time) are anaesthetized with a single intraperitoneal injection of 100 µL ketamine/xylazine anesthetic solution/20 g mouse using a sterile 1 mL insulin syringe. The depth of anesthesia is confirmed using the toe pinch method, which may take 5–7 min. Note: Each cage usually carries 5 mice. The above dosage will keep each mouse asleep for at least an hour, which is sufficient time to complete the entire stereotactic surgery process in five anesthetized mice.

#### 3.1.4. Preparation of the Biosafety Hood

The induction of anesthesia in step 2 can take up to 5–7 min. During this time, Section 3.1.3, Section 3.1.4, Section 3.1.5 and Section 3.1.6 are completed. The biosafety hood is disinfected using the same disinfection procedure as for the working bench: first with 10% bleach and then with 70% isopropyl alcohol, and allow the biosafety hood to dry. Note: This protocol involves the simultaneous use of two frames for the surgery of two mice; thus, a 6 ft biosafety hood is used.

#### 3.1.5. Setting Up the Stereotactic Frames

Before placing the frames inside the hood, their surfaces outside the hood at the workbench are disinfected with 10% bleach/Clorox and wiped with clean paper towels. Then, two stereotactic frames are placed inside the hood, leaving about 1.5 feet between them. This spacing allows for the safe and efficient handling of both frames during the surgery. Next, the frames are wiped with 70% isopropyl alcohol and allowed to dry completely. A heating pad is placed on each frame and turned on. Note: (i) The heating pad’s temperature is controlled by a heat regulator, so there is no risk of accidental overheating. Having a heating pad is critical because the frame stage can get cold under continuous laminar airflow, lowering the mouse’s body temperature; and (ii) two stereotactic frames (one mouse per frame) can be conveniently used simultaneously for stereotactic surgeries in two mice, allowing for the completion of surgeries in five mice within an hour.

#### 3.1.6. Setting Up Hamilton Syringe into the Frame

**Figure 1:**
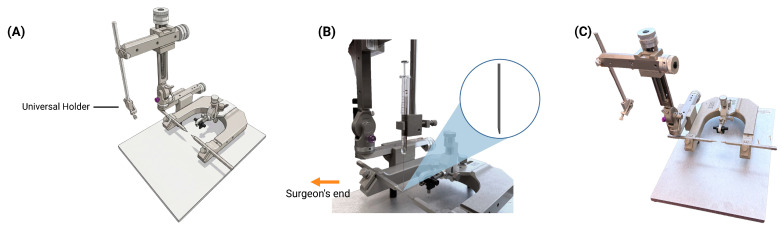
Stereotactic frame setup for intracranial procedures. (**A**) Stereotactic instrument used for the implantation of tumor cells, inoculation of oncolytic virus, and rechallenge study; (**B**) direction of needle on the stereotactic frame before surgery, and (**C**) keeping the universal needle holder away from the surgical stage before placement of the mouse on the frame.

The Hamilton Syringe is placed into the Model 1772 Universal Holder in such a way that the needle bevel opening is facing the surgeon and the scale up to 3 μL is clearly visible from the surgeon’s site (see Figure 1A,B). The screw is tightened to secure the Hamilton syringe in the Universal Holder and rotate the Universal Holder clockwise to 180° (90° from the base of the frame; see Figure 1C) to allow room for restraining the mice into the frame. The same process is repeated with the Hamilton syringe for the second frame.

#### 3.1.7. Disinfection of Hamilton Syringes/Needles

The syringes/needles are disinfected in three steps: (i) 10 µL 70% isopropyl alcohol is drawn into the syringe while keeping the tip of the needle approximately one inch submerged in the alcohol solution contained in a sterile 1.7 mL microcentrifuge tube. The plunger is moved up and down several times. (ii) The same process is repeated using sterile distilled water to remove any residual alcohol. (iii) Finally, the process is repeated using sterile PBS, then the PBS expelled from the syringe. Note: During all three disinfection steps, it should be ensured that there are no air bubbles in the syringe. If bubbles are observed, specifically in the final step, continue moving the plunger up and down until they are completely removed.

#### 3.1.8. Restraining of Mice on the Stereotactic Frame

**Figure 2:**
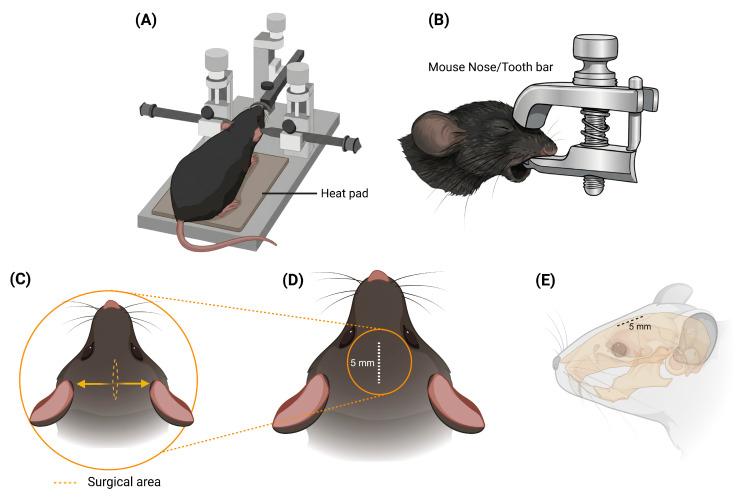
Surgical incision planning for triple-survival stereotactic procedures in mice. (**A**) Mouse positioning on the stereotactic frame for intracranial surgery, ear bars are inserted into the external auditory canals to stabilize the head; (**B**) mouse nose/tooth bar provides additional immobilization; (**C**) disinfection procedure of the surgical area; (**D**) dorsal view of the planned midline incision; and (**E**) lateral view of the incision trajectory.

Once anesthesia is established (as confirmed by the toe-pinch method in Section 3.1.2), the mouse is transferred to the pre-warmed heat pad placed on the stereotactic frame. One drop of mineral oil ointment is applied to each eye to prevent corneal drying. Then, the mouse is secured in the stereotactic frame by first positioning the left ear bar into the left ear, followed by the right ear bar into the right ear (Figure 2A). During ear bar placement, a subtle “cracking” sensation may be felt, indicating correct engagement of the ear bars within the external auditory canals. After securing the ear bars, the central incisors are positioned into the tooth holder of the Mouse 926-B Mouse Nose/Tooth bar Assembly of the stereotactic frame, as depicted in Figure 2B.

### 3.2. Preparation of the Surgical Site

Proper aseptic preparation of the surgical site is essential to reduce the risk of infection and ensure animal welfare.

#### 3.2.1. Disinfection of the Surgical Site

The surgical site is prepared under aseptic conditions by thoroughly disinfecting the scalp. First, a povidone iodine swab stick is applied to the surgical area, starting at the center of the surgical site and moving outward in a circular motion toward the surrounding area. Next, the area is cleaned using three successive cycles of 70% isopropyl alcohol. For each cycle, a fresh piece of gauze soaked in alcohol is used and wiped from the center outward, completing one full pass per cycle (Figure 2C). The disinfected area is allowed to dry completely before proceeding with surgery. Note: Hair removal is not necessary for this procedure, as it may increase the risk of microabrasions and skin irritation.

#### 3.2.2. Administration of Local Anesthesia

Local anesthesia is administered at the incision site to minimize pain during the surgical procedure. To do that, the skin is gently lifted at the intended incision site (bregma region) using forceps, and (to avoid excessive tissue pressure) ~30–50 µL/mouse of 0.25% bupivacaine solution slowly injected subcutaneously (Table 1) using a 1 mL insulin syringe.

### 3.3. First Survival Surgery: Intracranial Implantation of GSCs

The first survival stereotactic surgery is performed to establish an intracranial glioblastoma model by precisely implanting GSCs into the brain parenchyma. Accurate placement of GSCs at specified stereotactic coordinates is essential for consistent tumor development and the reliable assessment of downstream therapeutic interventions.

#### 3.3.1. Primary Incision

Using a sterile scalpel, a ~5 mm midline incision is made over the bregma to expose the skull (Figure 2D,E). Then, the skin is gently separated with a sterile cotton swab to clearly visualize the surgical field and identify the bregma.

#### 3.3.2. Determine the Injection Site

The point of injection is identified, which is 2.2 mm lateral to bregma (or sagittal suture) on the right side of the midline and 1 mm anterior toward the coronal suture. Note: If the visualization of the bregma area is obscured by bupivacaine solution or bleeding, the area should be gently cleaned and dried with a sterile cotton bud.

#### 3.3.3. Drilling at the Stereotactic Coordinate

**Figure 3:**
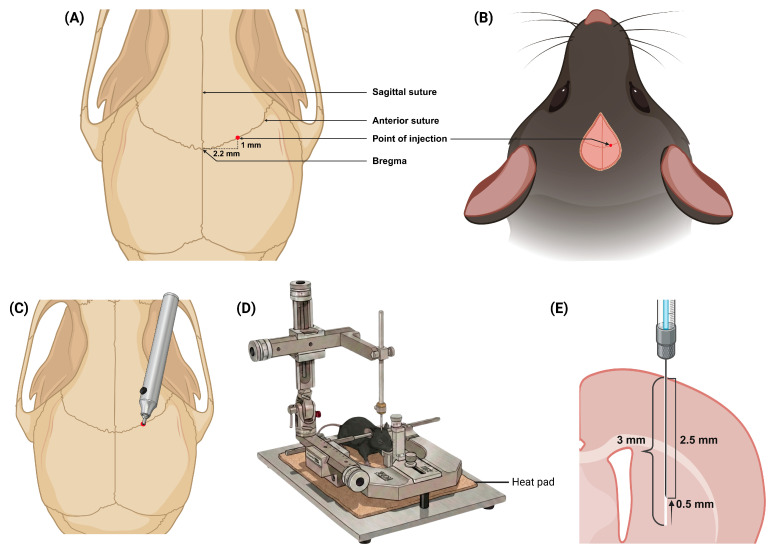
Stereotactic targeting of the mouse brain. (**A**) Stereotactic coordinates and reference sutures used to locate the surgical site; (**B**) dorsal view of the incision site relative to the cranial landmarks; (**C**) dorsal view of the skull showing the drilling site; (**D**) mouse secured in the stereotactic frame with all restraints in place before initiation of surgery; and (**E**) needle insertion trajectory and injection depth, indicating the final position of the needle tip during infusion.

Using an Ideal Micro-Drill with a bit size of 0.6 mm, a burr hole is created at the identified site, not exceeding 0.6 mm in diameter. (Figure 3A–C). Notes: (i) Extreme care should be taken to drill only through the skull without penetrating the meninges or brain tissue, as this may cause intracranial hemorrhage and compromise outcomes. (ii) It should be ensured that at least two functional micro-drill units are available (primary and backup), with fully charged batteries prior to surgery to avoid procedural delays or interruptions.

#### 3.3.4. Syringe Loading of 005 GSCs

Cells will settle to the bottom of the microcentrifuge tube, so the bottom of the tube is gently flicked 5–7 times with the index finger to ensure homogenous distribution immediately before withdrawing the cell suspension into the syringe. Before loading the syringe, note that residual PBS (from the final rinse step in Section 3.1.6) in the needle may dilute the initial cell suspension and introduce variability, especially for the first injection (i.e., the first mouse may receive a mixture of residual PBS and 005 GSCs). To minimize this, the plunger is repeatedly moved up and down while the needle tip is immersed in the cell suspension to flush residual fluid homogeneously into the suspension. Then, 3 µL of the cell suspension is aspirated into the Hamilton syringe. The cells are kept on ice.

#### 3.3.5. Intracranial Injection of 005 Cells

The needle is positioned directly above the burr hole using the stereotactic arm (Figure 3D). The starting position is defined as the point where the needle tip just enters the hole while the bevel remains visible. The needle is advanced 3 mm into the striatum, then withdrawn 0.5 mm to create an empty space to facilitate smooth injection and reduce backflow (Figure 3E). Then, the cell suspension is injected slowly at a rate of 0.1 µL/s (~30 s total), as rapid infusion can increase intracranial pressure and reduce reproducibility. After injection, the needle is kept in place for at least 3 min to allow for the cells to settle. During this waiting period, the surgical procedure is proceeded with on a second mouse using the second stereotactic frame to improve efficiency while maintaining consistency.

#### 3.3.6. Needle Withdrawal

The needle is slowly withdrawn (~0.5 mm every 12 s; 60 s total). Immediately, the needle is wiped with dry surgical gauze before reuse to prevent cross-contamination and the unwanted transfer of blood or tissue from one mouse to the next, as we are using the same needle repeatedly throughout the experiment. There is no PBS wash between injections.

#### 3.3.7. Sealing and Closure

The tip of a sterile cotton swab is used to pick up a small amount of bone wax and gently apply it directly over the burr hole, pressing carefully to fully seal the opening. The incision is closed with a single simple interrupted 5-0 nylon suture. Section 3.6 describes postoperative care.

### 3.4. Second Survival Surgery: Intratumoral Injection of oHSV

Following tumor establishment, a second survival stereotactic surgery is performed to deliver therapeutic agents directly into the tumor. The rationales for this follow-up second survival surgery (i.e., virus injection directly into intracranial tumors) are as follows: (i) local treatment maximizes tumor accumulation compared to systemic virus administration [38], as local virus treatment requires lower viral dose for tumor accumulation than systemic treatment, because complement or preexisting anti-virus serum antibodies limit anti-cancer efficacy of systemically delivered oncolytic viruses [39]; (iii) because OVs have been engineered for tumor-specific replication, local application confines virus replication and virus spread only within the tumor cells and tumor microenvironment as demonstrated by the absence of viral DNA in liver, spleen and peripheral blood [40]; and (iv) several OVs in the clinic are being administered directly into the tumor and have proven to be safe in aotus nonhuman primates and humans [11,41,42,43,44,45,46,47].

The timing of the second survival surgery for oHSV injection depends on the experimental objective. For studies related to therapeutic intervention, the second surgery is typically performed around day 8 post-tumor implantation [10,14,16,21]. For tumor tissue analysis or mechanistic studies, later time points (e.g., day 17–18 or 24) may be used to allow tumor progression [14,21]. In some experimental designs, oHSV can be injected intracranially multiple times (e.g., days 8 and 12) depending on the treatment strategy [10]. Here, in this protocol, we described a single intratumoral administration of genetically engineered G47Δ-IL12 oHSV to induce tumor cell killing and stimulate local antitumor immune responses. Direct intratumoral delivery of oHSV ensures precise localization while minimizing damage to surrounding tissue. Although this protocol describes the administration of the G47Δ-IL12 virus, the surgical procedure can be adapted for intracranial delivery of other OVs, biologics, or chemotherapeutic agents.

#### 3.4.1. Surgical Approach

The same preoperative care and pain management procedures are followed as described for the first survival surgery in Section 3.1 and Section 3.2. The second survival stereotactic surgery is performed 1–4 weeks after intracranial tumor implantation, depending on tumor growth and study design. The same stereotactic coordinates are used as those for tumor cell implantation (Section 3.3) to ensure accurate targeting.

#### 3.4.2. Reopening the Surgical Site and Wax Removal

The previous incision is opened using a sterile scalpel and expose the skull. The original burr hole is located and used as the entry point for the intratumoral delivery of oHSV. The bone wax is carefully removed and the drilled hole sealed by gently lifting and peeling it off with the tip of a sterile scalpel blade. Note: This step is performed slowly and with minimal pressure to avoid damaging the skull or underlying brain tissue.

#### 3.4.3. OHSV Loading and Needle Positioning

The viral suspension is loaded into the syringe, moving the plunger up and down to eliminate air bubbles and ensure uniform filling. The needle is inserted through the existing burr hole using the same stereotactic coordinates and depth as in the initial tumor implantation (Section 3.3). Consistent positioning ensures accurate delivery into the established tumor mass.

#### 3.4.4. Intratumoral Injection

The viral solution is slowly injected at a controlled rate of 1 µL per 15 s (approximately 30 s total). Slow infusion minimizes tissue disruption, reduces reflux along the needle track, and promotes even distribution within the tumor. After injection, the needle is kept in place for 2–3 min to allow the solution to diffuse properly and prevent backflow during withdrawal.

#### 3.4.5. Closure and Postoperative Care

The needle is withdrawn slowly as in Section 3.3.6 and then the burr hole resealed with bone wax and close the incision site following the procedures described in Section 3.3.7. Section 3.6 describes postoperative care.

### 3.5. Third Survival Surgery: Intracranial Rechallenge with GSCs

In most cancer patients, current standard-of-care treatments, including surgery, radiation, chemotherapy, and immunotherapy, rarely achieve complete and durable cures. Even when tumors are controlled, long-term immunological memory is often inadequate or absent. As a result, tumors may recur months or years later, frequently with increased resistance to conventional therapies [3]. Therefore, it is essential to determine whether treatments that eliminate tumors in preclinical models also induce durable immune memory capable of preventing tumor recurrence.

Antitumor immune memory in ‘tumor-free’ long-surviving mice can be evaluated by intracranial rechallenge with the same tumor cells in the contralateral (opposite) hemisphere, as previously described (i.e., third survival surgery) [14]. In this approach, GSCs were implanted intracranially into the contralateral (opposite) hemisphere of mice [14]. If effective antitumor immune memory has developed following the initial treatment, rechallenged mice will resist tumor formation or show significantly delayed tumor growth. For example, complete memory protection was observed in mice treated with a triple combination therapy (G47Δ-IL12 plus dual immune checkpoint blockade) [14]. In contrast, tumor development following rechallenge indicates insufficient or absent memory protection. The third survival stereotactic surgery is performed in tumor-free long-term surviving mice at a predetermined experimental time point following treatments, using the hemisphere contralateral to the initial tumor implantation and treatment sites.

#### 3.5.1. Preoperative Preparation

The same perioperative procedures is followed as described in Section 3.1 and Section 3.2, including anesthesia, aseptic technique, and pain management.

#### 3.5.2. Determine the Injection Site at the Contralateral Side

After confirming adequate anesthesia, a ~5 mm midline incision is made to expose the skull. The injection site is identified in the contralateral hemisphere at 2.2 mm lateral to bregma (or the sagittal suture) and 1 mm anterior to the coronal suture.

#### 3.5.3. Tumor Cell Implantation and Wound Closure

All subsequent steps are performed, including skull drilling, intracranial injection of GSCs, controlled needle withdrawal, sealing of the burr hole with bone wax, and wound closure with nylon sutures, according to the procedures described for the first survival surgery (Section 3.3). The injection of a larger cell number (i.e., 2–5 fold) in the same volume may be considered to ensure the development of immune memory.

Section 3.6 describes postoperative care.

### 3.6. Postoperative Care

Standardized postoperative care should be applied after each surgery to ensure consistent recovery, minimize variability, and maintain animal welfare throughout the study.

#### 3.6.1. Immediate Recovery

Single housing is provided during the immediate recovery period. The mouse is removed from the stereotactic frame and placed in a clean cage on a warm bench surface. A heat source (e.g., heat lamp) is used, positioned away from the animal to prevent overheating. The mouse is monitored continuously until it is fully awake and ambulatory.

#### 3.6.2. Analgesia

A new cup of carprofen oral gel is added in the cage (1 cup/cage) for postoperative pain management. Additionally, carprofen (6 mg/kg/day, subcutaneously) is administered for postoperative analgesia if clinical signs of discomfort are observed (e.g., hunched posture, restlessness, or discharge from the surgical site). Postoperative pain management is continued for up to 72 h post-surgery, as needed. Note: If analgesia is insufficient or unexpected complications arise, consult veterinary staff and provide supportive care as recommended. Ethiqa XR, an extended-release buprenorphine, could be an alternative that lasts 72 h with a single injection, reducing post-surgery handling of mice.

#### 3.6.3. Monitoring

Animals are monitored at least twice daily during the postoperative period. Body weight, activity level, grooming behavior, posture, and neurological status are recorded. Food is placed on the cage floor to ensure easy access for mice with reduced mobility or coordination.

#### 3.6.4. Suture Removal

Nylon sutures are removed once the incision site has healed, typically occurring within 5–7 days post-surgery. Note: Mice may remove sutures earlier due to grooming or scratching.

### 3.7. Study Endpoint and Euthanasia Criteria

#### 3.7.1. Study Endpoint

Representative mice from each experimental group undergoing survival neurosurgeries are humanely euthanized at defined study endpoints to evaluate tumor development and assess the antitumor efficacy of oHSV-based therapy.

#### 3.7.2. Humane Endpoints

Animals showing signs of physical distress should be euthanized immediately to minimize suffering. Criteria include ≥20% body weight loss, lethargy or reduced responsiveness, neurological symptoms (e.g., seizures, circling, paralysis), labored breathing, infection or poor wound healing, or any condition that compromises animal welfare, as determined by trained personnel or veterinary staff.

#### 3.7.3. Euthanasia Methods

Moribund animals or those with severe neurological impairment should be humanely euthanized in accordance with established guidelines. Euthanasia may be performed using inhalation of 100% CO_2_ in a controlled chamber, following recommendations from the American Veterinary Medical Association. Perform a secondary physical method (e.g., cervical dislocation or bilateral thoracotomy) to ensure death. Confirm death by the absence of respiration and heartbeat before tissue collection or disposal. Note: Death is not considered an experimental endpoint.

## 4. Expected Results

**Figure 4:**
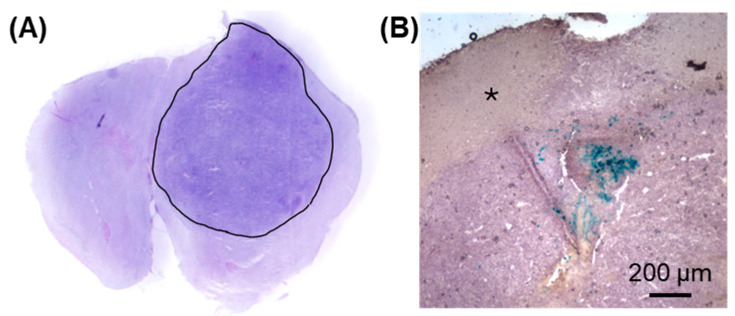
(**A**) A representative mouse brain section showing an intracranial 005 tumor (solid line) in the right cerebral hemisphere. 005 GSCs (2 × 10^4^) were implanted into the brains of C57BL/6 mice according to the protocol described for the first survival surgery (see Section 3.3). Mice were followed for survival; brains were collected (*n* = 3), fixed in formalin, embedded in paraffin, stained with hematoxylin and eosin (H&E), and the histological slides were scanned. (**B**) In C57BL/6 mice, 005 GSCs (5 × 10^5^) were implanted on day 0. G47∆-IL12 (1 × 10^6^ pfu/mouse) was injected intratumorally on day 19 following the protocol described for the second survival surgery (see Section 3.4). 36 h after virus injection, mice were perfused with ice-cold PBS, followed by fixation in a brain fixative. Brains were stained for X-gal, frozen, sectioned, and further stained for X-gal and counterstained with hematoxylin. X-gal stains virus-infected cells due to LacZ expression. Bar = 200 μm; * non-tumor areas (see details in Saha et al. 2017 [14]).

Following completion of the protocol, mice are monitored longitudinally until they reach predefined humane endpoints, which typically occur around 35 days post-implantation in mock-treated groups, depending on tumor growth kinetics and treatment interventions [14,21]. The expected outcomes of this protocol include robust and reproducible intracranial tumor establishment following the first survival surgery (Figure 4A,B), accurate intratumoral localization of oHSV after the second survival surgery, and effective rejection of rechallenge tumor cells implanted during the third survival surgery, reflecting the development of immunological memory responses as demonstrated previously [14]. Histological analyses are expected to confirm tumor formation and distribution of oHSV at the injection site (Figure 4A,B). Under standardized conditions, tumor establishment rates are expected to be highly consistent across animals when the protocol is performed as described.

Animals are closely monitored for clinical signs of disease progression and distress, which commonly arise from intracranial tumor burden. These include body weight loss (≥15%), reduced mobility, hunched posture, lethargy, persistent recumbency, rough or unkempt coat, labored breathing, dehydration, and neurological symptoms such as circling, head tilt, limb paralysis, seizures, or hydrocephalus. Reduced food or water intake and decreased responsiveness to external stimuli are also considered indicators of declining health status. Animals showing severe or persistent symptoms are euthanized immediately to minimize suffering. Additional criteria include abnormalities lasting longer than 24 h, such as inactivity or hyperactivity, piloerection, abnormal vocalization, or anorexia. While some mild or transient symptoms may occur during tumor progression, these are not expected to significantly impact overall animal health. Supportive care, including hydration gel supplementation or veterinary intervention, may be provided when appropriate to maintain animal welfare.

Long-term survivors, defined as tumor-free mice following successful therapeutic intervention, are maintained for extended observation to evaluate durable treatment responses and immunological memory [14]. These animals are typically euthanized at a time point at least twice the survival duration of mock-treated controls, ensuring sufficient time to assess long-term outcomes. Rechallenged mice are monitored using the same humane endpoint criteria, and resistance to tumor formation or delayed tumor growth serves as a functional readout of effective immune memory [14].

## 5. Discussion

Compared with previously reported stereotactic protocols, which typically involve single or dual surgical interventions, this triple-survival workflow uniquely integrates tumor establishment, therapeutic delivery, and immune memory assessment within a single animal. By combining intracranial implantation of GSCs, intratumoral delivery of oHSV, and contralateral tumor rechallenge, this approach allows simultaneous assessment of both primary tumor control and the development of durable antitumor immunity. This integrated design represents a significant advantage over conventional models, in which these parameters are typically evaluated in separate experimental systems.

The precise intracranial delivery of tumor cells and therapeutic agents is critical for the success of this model. Stereotactic surgery remains the gold standard for achieving accurate and reproducible targeting within the brain. The use of consistent stereotactic coordinates and reuse of the same burr hole for tumor implantation and viral delivery minimizes disruption to the brain parenchyma, reduces procedural variability, and promotes faster postoperative recovery. These factors collectively improve experimental consistency and reproducibility across animals and studies.

However, repeated survival surgeries introduce potential limitations, including cumulative surgical stress, prolonged exposure to anesthesia, and an increased risk of localized inflammation. These factors may influence tumor biology or immune responses and should be considered when interpreting experimental outcomes. In addition, deviations in stereotactic targeting, such as injecting at different coordinates across procedures, can lead to increased tissue damage, variability in tumor growth, and inconsistent therapeutic delivery. Maintaining precise and standardized surgical techniques is therefore essential to minimize bias and ensure reproducibility.

Intratumoral delivery of oHSV offers several advantages over systemic administration. Local delivery enhances antitumor efficacy by concentrating the therapeutic agent within the tumor microenvironment while reducing systemic exposure. It also allows for lower viral doses, minimizes the impact of neutralizing antibodies and complement-mediated inactivation, and supports tumor-specific viral replication with limited off-target distribution. These features contribute to an improved safety profile and enhanced therapeutic potential. Furthermore, this protocol is adaptable and can be extended to the delivery of other viral vectors, transgenes, biologics, or chemotherapeutic agents requiring intracranial administration.

The use of intracranial tumor models is essential for translational relevance, as these models closely recapitulate the anatomical, physiological, and immunological characteristics of human brain tumors. Unlike subcutaneous models, intracranial models capture clinically relevant features such as tumor–brain interactions and neurological symptom progression. Consequently, monitoring relies on behavioral and physiological parameters rather than tumor size alone.

Importantly, death is not considered an experimental endpoint in this protocol; instead, strict humane endpoint criteria outlined in Section 3.7 are applied and rigorously enforced to minimize animal distress. All surgical procedures are performed under appropriate anesthesia, with comprehensive pre- and postoperative care to reduce pain and distress. Although surgical interventions and injections may cause transient discomfort, these effects are generally mild and manageable. Based on prior experience, adverse effects associated with oHSV therapy at the specified dose and schedule are rare.

Overall, this triple-survival stereotactic surgery model provides a powerful and versatile platform for evaluating novel therapeutic strategies in GBM, particularly those aimed at studying memory protection. This integrated approach addresses a critical gap in preclinical modeling by enabling simultaneous assessment of therapeutic efficacy and immune memory within a single experimental system, and may facilitate the development of more effective and long-lasting treatments for patients with brain tumors.

## 6. Conclusions

This triple-survival stereotactic neurosurgical protocol provides a robust and reproducible platform for studying GBM progression, therapeutic response, and long-term antitumor immunity within a single experimental framework. By integrating intracranial tumor establishment, precise intratumoral delivery of oHSV, and contralateral tumor rechallenge, this approach enables comprehensive evaluation of both tumor eradication and immune memory. The use of consistent stereotactic techniques and standardized perioperative care ensures high reproducibility and minimizes variability across experiments. Importantly, this model is adaptable to a wide range of therapeutic agents and can be extended to other intracranial tumor systems, including breast cancer brain metastasis models. As such, it provides a scalable and translationally relevant platform for advancing therapies that achieve both tumor control and durable immune protection in GBM and other brain malignancies.

## Figures and Tables

**Table 1:** Anesthetic, analgesic, and supportive agents used for the triple-survival stereotactic surgery in mice.

Agent	Dosage	Route	Schedule	Volume
Bupivacaine	0.25%	Subcutaneously around bregma	Once	30—50 µL
Xylazine	10 mg/kg	Intraperitoneal	Once (in combination with ketamine)	100 µL combination mixture/20 gm body weight
Ketamine	100 mg/kg	Intraperitoneal	Once (in combination with xylazine)	
Carprofen	10 mg/kg/day	Oral dose	One day before surgery	One cup/cage
Carprofen	6 mg/kg/day	Subcutaneous	After surgery	As appropriate

## Data Availability

The data supporting the procedures and findings of this study are contained within the article.
